# Gut instincts in neuroimmunity from the eighteenth to twenty-first centuries

**DOI:** 10.1007/s00281-022-00948-2

**Published:** 2022-07-04

**Authors:** Mytien Nguyen, Noah W. Palm

**Affiliations:** 1 Department of Immunobiology, Yale University School of Medicine, 10 Amistad Street, New Haven, CT 06520 USA; 2 Aligning Science Across Parkinson’s (ASAP) Collaborative Research Network, Chevy Chase, MD 20815 USA

**Keywords:** Neuroinflammation, Gut-brain axis, Microbiota, Microbial metabolites, Neurodegenerative diseases

## Abstract

In the past two decades, work on the microbiota-gut-brain axis has led to a renewed appreciation for the interconnectedness between body systems in both clinical and scientific circles. In the USA alone, millions of adults are burdened with non-communicable chronic diseases whose putative etiologies were previously thought to be restricted to either the gut or brain, such as inflammatory bowel disease, irritable bowel syndrome, Parkinson’s and Alzheimer’s disease, and autism spectrum disorder. However, the recent explosion of research into the impacts of the gut microbiome on diverse aspects of human health has revealed the potentially critical importance of reciprocal interactions between the gut microbiota, the immune system, and the brain in diverse diseases and disorders. In this review, we revisit the history of gut-brain interactions in science and medicine, which dates back to at least the eighteenth century, and outline how concepts in this field have shifted and evolved across eras. Next, we highlight the modern resurgence of gut-brain axis research, focusing on neuro-immune-microbiota interactions and recent progress towards a mechanistic understanding of the diverse impacts of the microbiome on human health. Finally, we offer a forward-looking perspective on the future of microbiota-gut-brain research, which may eventually reveal new paths towards the treatment of diverse diseases influenced by the complex connections between the microbiota and the brain.

## Introduction

The recent explosion of interest in the role of the gut microbiota in human health has led to a growing interest in the so-called microbiota-gut-brain axis. However, the first studies of the gut-brain axis date back more than three centuries. In this review, we explore the saga of the gut-brain axis over the centuries, with a focus on microbiota-neuroimmune communication. We begin by outlining the clinical and scientific conceptualization of the gut-brain axis in the 1700s before reviewing modern investigations of the underlying mechanisms governing gut-brain communication. Finally, we speculate on how our understanding of the myriad links between the gut and brain may shift in the future.

## Gut-brain axis: a historical perspective

**Fig. 1:**
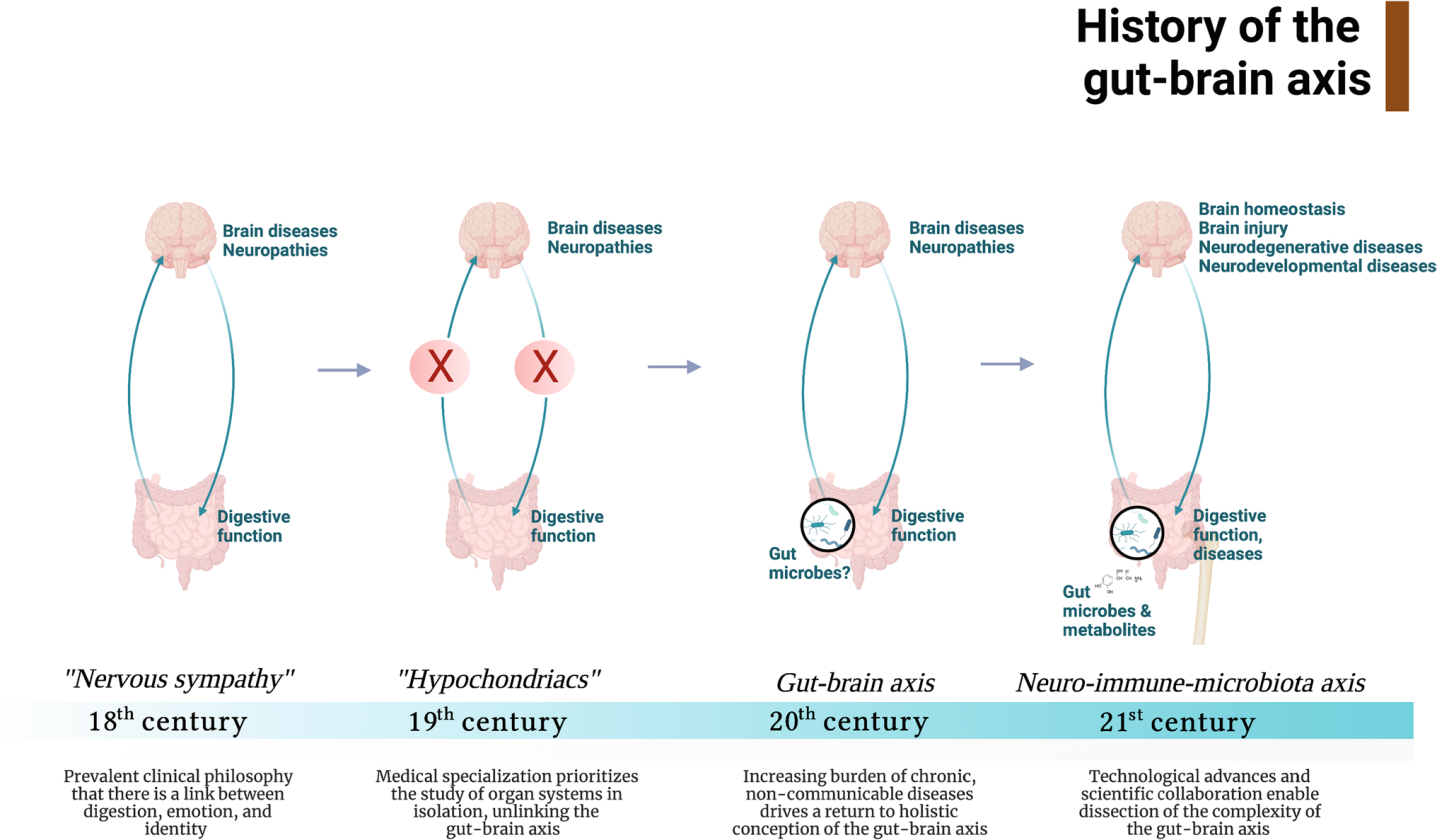
History of the gut-brain axis: trends in clinical and scientific understanding of the gut-brain axis from the eighteenth to twenty-first centuries. In the eighteenth century, the gut-brain axis was conceptualized by most clinicians and scientists as two organs that constantly communicate. However, this view shifted in the nineteenth century as medicine became increasingly specialized. In the twentieth century, a resurgence of interest in gut-brain communication emerged as the USA faced an increasing burden of chronic, non-communicable diseases. The modern twenty-first century understanding of the gut-brain axis is characterized by an appreciation for its complexity, the emerging fields of the gut microbiome and neuroimmunology, and the increasing promise of gut-brain interventions as novel therapeutic approaches to treat neurological disease

Scientific and clinical perspectives on the gut-brain axis have historically cycled between holistic and individualized approaches. Early descriptions of the gut-brain connection can be traced back at least three centuries. In the eighteenth century, physicians’ conception of the connection between the gut and brain was primarily holistic. It centered around the idea that digestion, emotions, and identity are linked and that individuals’ digestive functions influence both mind and mood [1–3]. Furthermore, this connection was bidirectional: the mind affects digestive function, and digestive function influences the mind (Fig. 1). In 1765, Robert Whytt, a Scottish physician, introduced the concept of *nervous sympathy* in which all internal body organs, including the gut and the brain, are interconnected by a single communication network [4]. Nervous sympathy thus reflects how eighteenth and nineteenth century scientists and physicians conceptualized the reciprocal nature of the gut-brain axis—that is, an unhealthy digestive system causes an abnormal mind.

Beginning in the late nineteenth century and continuing into the twentieth century, increased specialization in medicine ushered in a Golden Age of medical discovery [5]. This enabled rigorous and focused dissection of specific health problems, leading to more in-depth clinical science and medical breakthroughs such as the polio vaccine in 1955 [6]. However, partitioning different organs to various clinical specialties set back the clock on the holistic approach to understanding the gut-brain connection that was dominant in the eighteenth century. For example, nineteenth-century physicians often dismissed patients with gastrointestinal issues of unclear etiology as hypochondriacs. And, even in the 1970s, physicians would often diagnose patients that exhibited gastrointestinal symptoms with no apparent organic cause with psychiatric rather than gastrointestinal illness [7].

The development of the germ theory of disease by Louis Pasteur and Robert Koch ushered in a new era of microbe-focused research and innovation [8]. The burden of communicable diseases decreased dramatically beginning in the mid-nineteenth century due to improvements in sanitation hygiene as well as the development of vaccines and antibiotics. At the same time, the burden of non-communicable diseases rapidly increased [9]. Unlike infectious diseases, non-communicable conditions were more lifestyle-driven and often chronic [5, 9]. Faced with this new challenge, clinicians and scientists once again began to approach the body holistically. This transition was marked by a steady progression of research seeking to dissect how the gut affects central nervous system functions and vice versa. For example, surgeons observed that post-operative jejunoileostomy patients experienced episodic central nervous system symptoms, such as slurred speech and confusion, which sometimes recurred months after their operation [10]. These observations challenged previous conceptions that the small intestine and the colon were merely tubes for waste materials [7].

Indigenous microbial inhabitants of the gut, now known as the microbiota, also came into focus as a potential key to understanding the gut-brain connection. One of the first proponents of this concept was Russian embryologist Elie Metchnikoff, who proposed that probiotic bacteria found in yogurt could promote health and delay senility more than a century ago [11]. This idea remained largely dormant for many decades, before reemerging in the mid to late twentieth century when multiple research groups began to explore the impact of alterations in the gut microbiota on mammalian phenotypes in rodent models. Ian Rowland in the UK found that toxic concentrations of mercury and associative neurotoxic symptoms were more pronounced in rats treated with antibiotics [12]. Antibiotic administration in rats also altered biogenic amine concentrations in plasma [13, 14]. The generation of germ-free rats that lack all indigenous microbes led to further targeted studies of the gut-brain axis and numerous studies in the late-twentieth century revealed that diverse CNS phenotypes differed between GF and conventional animals. Linda Hegstrand and R. Jean Hine from the William S. Middleton Memorial Veterans Hospital in Wisconsin found that conventional rats had higher hypothalamic histamine levels than germ-free (GF) rats [15]. Furthermore, 1,3-dinitrobenzene administration induced ataxia in GF but not conventional rats [16]. These studies laid the foundation for a growing consensus that gut-brain connections are critical for human health. At the turn of the century, scientists began to examine whether specific commensal bacteria might even prevent or reverse neuropathology. In 1965, following on earlier studies of the probiotic *Lactobacillus* by Minoru Shirota and Elie Metchnikoff in intestinal health [11], William Macbeth of Harvard Medical School performed one of the first experiments testing the impact of probiotics on the brain when he successfully treated two patients with hepatic encephalopathy with *Lactobacillus acidophilus* [17].

In the late twentieth century, advances in understanding the connection between the gut and the brain were spurred on by the establishment of the new field of neuroimmunology, which began to challenge the traditional assumption that the brain is segregated from the immune system (Fig. 1). A series of pioneering studies by early proponents of this field demonstrated a critical role for T lymphocytes in maintaining brain homeostasis, injury repair, and resolution of neuroinflammation [18–24]. The shift of clinical research towards a more team-based and multidisciplinary approach in the late twentieth century [25] led to mechanistic insights into the gut-brain axis and the immune system’s role in these interactions. Patrick Dougherty’s lab in the 1980s found that the bacterial product 6–0-stearoyl-muramyl dipeptide (MDP) could attenuate opiate withdrawal severity in a dose-dependent fashion when injected directly into the brain [26, 27]. Sylvain Nadeau and Serge Rivest at Laval University found that myeloid-derived cells in the brain express the LPS receptor CD14, indicating that brain-resident myeloid cells may sense peripheral bacterial products [28]. At the same time, a few studies began to indicate a critical role for the immune system in mediating the bidirectional communication between the gut and the brain. Specifically, Baciu et al. found that tuberomammillary lesions dramatically reduced the phagocytic activity of circulating immune cells [29]. These early indications of neuro-immune-microbiota connections paved the way for the gut-brain-microbiome boom that would occur in the twenty-first century. During this same era, advances in next-generation sequencing enabled facile assessments of microbiota composition independent of microbial culture via 16S rRNA gene sequencing. Many early research efforts using these technologies focused on identifying individual causative agents of non-communicable human diseases; however, after failing to find individual pathogens responsible for these diseases, the field largely converged on the modern holistic view of microbiota-mediated impacts on host health [30].

## Modern understanding of neuro-immune-microbiota connections

Modern clinical conceptions of the neuro-immune-microbiota axis are, in essence, an homage to the eighteenth- and nineteenth-century holistic approaches to understanding the gut-brain connection. A preponderance of clinical and pre-clinical data underscore the consistent comorbidity between neuropsychiatric diseases and intestinal pathologies [31, 32]. Correspondingly, the gut microbiota has been found to modulate psychological outcomes, such as behavioral abnormalities in neurodevelopment and the anti-seizure effects of ketogenic diets [33, 34]. An explosion of recent studies has also provided insights into the role of the neuro-immune-microbiota axis in complex diseases such as ulcerative colitis, inflammatory bowel disease, Parkinson’s disease, and multiple sclerosis [35, 36]. Indeed, in the past 21 years, there have been 2.5 times as many publications in this field as compared to the past century altogether. Most recently, spurred on by the rapid growth of the microbiome field, mechanistic insights into the role of the immune system in the gut-brain axis have started to come into focus. Below, we offer a brief review of the current understanding of the neuroimmune-microbiota connection with a focus on the influence of bacteria and their metabolites on neurodegenerative disease.

### The classical role of microglia in neuroinflammation

Considered endogenous macrophages of the central nervous system, microglia are essential for tissue homeostasis in the brain. Microglial activation is central to the macrophage theory of depression, where neuroinflammation is a core contributor to abnormal depressive behaviors [37]. Microglia dysfunction is also prominent in other neuropathologies, including schizophrenia (increased microglial activity and density, elevated expression of proinflammatory cytokines) [38, 39], Parkinson’s disease (increased activation in the substantia nigra by alpha-synuclein, proinflammatory profile) [40, 41], Alzheimer’s disease (increased activation, synaptic remodeling) [42–44], and multiple sclerosis (increased activation profile and oxidative stress) [45, 46].

Building upon earlier experiments from the Rivest lab [28], multiple research groups found that GF mice and mice with a dysbiotic gut microbiota have abnormal microglia populations in the hippocampus, cortex, and cerebellum [47–49]. Microglia from GF mice are immature as compared to microglia from conventionalized mice [48], as characterized by higher *Ki67* and *Csflr* expressions and diminished capacity to produce a variety of chemokines and cytokines upon infection [48]. This “dysbiotic” microglial population ultimately leads to a reduced ability to fight against both systemic and local bacterial and viral infections [48]. Microglial morphology is also altered in GF mice and mice colonized with simple bacterial communities, with increased branching [48].

**Fig. 2:**
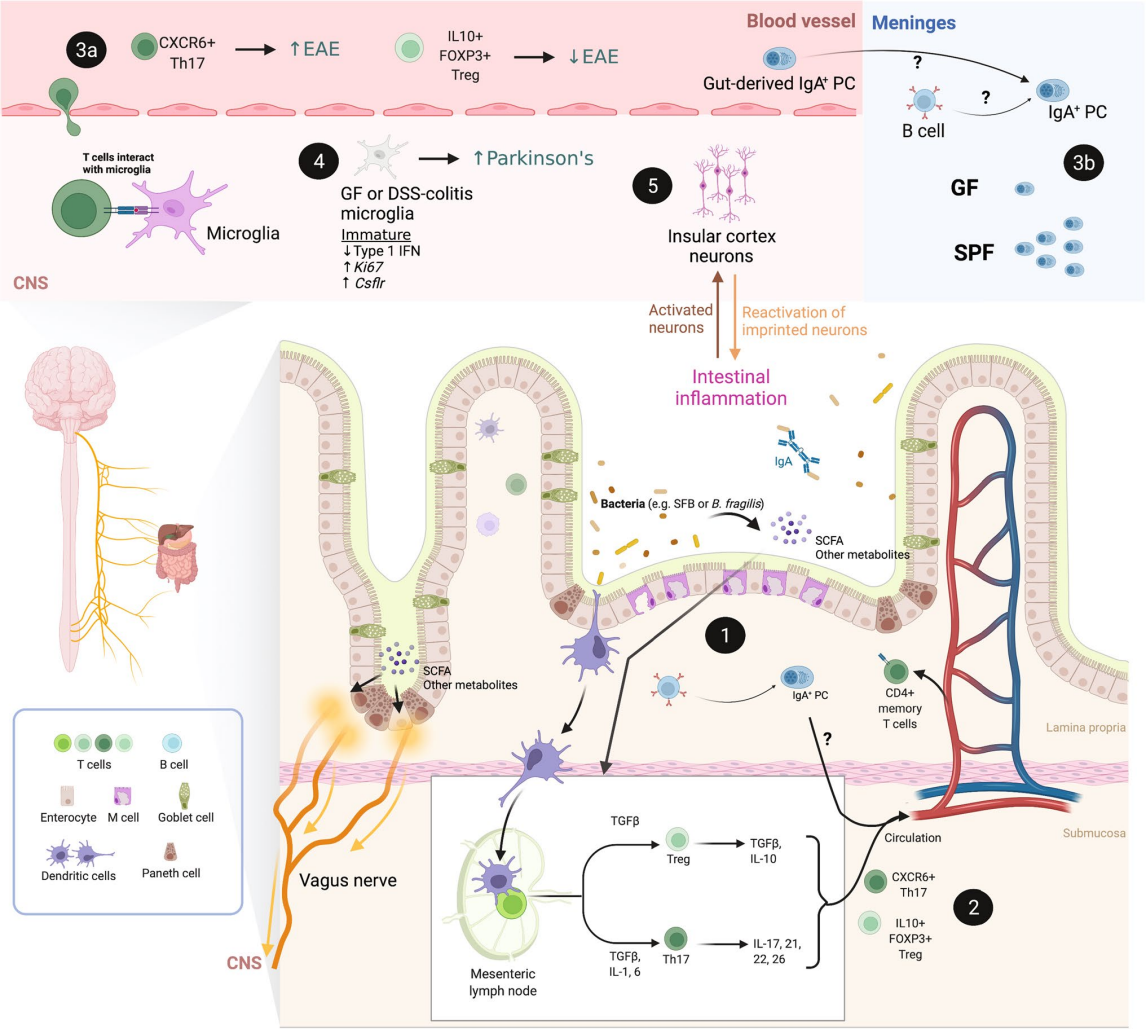
Mechanistic insights into the neuro-immune-microbiota axis. The role of microglia, T and B lymphocytes, and neurons in mediating interactions between the gut and the brain in homeostasis and disease. Modern techniques in microbial manipulation and sterilization (e.g., germ-free [GF] mice) and immunological advances enable precise dissection of the role of specific gut microbes and immune cells in modulating central nervous system (CNS) diseases. Gut microbes and their metabolites influence intestinal T and B cell activation and differentiation (1). A subset of intestinal T and B cells can circulate from the gut (2) to the meninges where they influence the local neuro-immune microenvironment by releasing cytokines (e.g., IL-17a, IL-10) and antibodies (e.g., IgA) that act on central neurons and microglia and protect against meningeal infection (3a: T cells; 3b: B cells). Dysbiotic and GF mice have altered microglia, which are immature and hyperproliferative (4). Lastly, accumulating evidence suggests that vagal and insular cortex neurons are critical in mediating the bi-directional communication between the gut and the brain and that insula neurons can retrieve and reactivate past inflammatory events (5). Abbreviations: EAE: experimental autoimmune encephalomyelitis; GF: germ-free; SPF: specific-pathogen-free; PC: plasma cells; CNS: central nervous system; SCFA: short-chain fatty acids; SFB: segmented filamentous bacteria

Altered microglial profiles in GF versus conventional mice can be at least partially explained by the effects of microbial-derived metabolites on microglia (Fig. 2). The gut microbiota produces thousands of unique small-molecule metabolites, some of which can accumulate systemically and reach extra-intestinal tissues, including the brain [35, 50]. Short-chain fatty acids (SCFAs), which are produced during microbial fermentation of dietary fiber, are among the best-studied microbial metabolites [50]. Reduced SCFA concentrations have been associated with multiple CNS pathologies, such as brain amyloidosis in Alzheimer’s disease [51]. Furthermore, SCFA administration restored microglial activation profiles and functions in GF mice [48]. Conversely, microbial-derived SCFA promoted microglial activation and enhanced motor dysfunction in a mouse model of Parkinson’s disease [52]. SCFAs also induced microglial production of neuroprotective IL-10 [53]. Beyond SCFA, other microbial metabolites such as indole and its derivatives have also been shown to influence microglial activation and neurotoxicity [54–57]. Microglial activation is also thought to be an initial step in the chemical-induced neurotoxic cascade [58]. Gut microbes that synthesize AhR agonists from dietary tryptophan (e.g. *Peptostreptococcus russellii*), also influence microglial activation by promoting TGFα production and modulating astrocyte activation and neuroinflammation [54–56].

Emerging evidence also suggests that microglial neuroinflammation in the central nervous system may result from microbiota-mediated priming via the peripheral immune system (Fig. 2). In mice treated with dextran sulfate sodium (DSS) to induce colitis, researchers observed parallel and synergistic inflammatory responses in the gut mucosa and the cerebral cortex, marked by increased expression of *IL6* and *iNOS* [59]. DSS-colitis ultimately led to microglial alterations characterized by increased activation and elevated cytokine levels, which mirrors the microglial phenotype in germ-free mice [48]. Potential priming of microglia by peripheral immunity is also supported by data from Parkinson’s disease mouse models. For example, DSS–induced intestinal inflammation led to accelerated brain neuropathology and motor dysfunction in a Parkinson’s disease model [60]. The age of onset of motor dysfunction was also significantly earlier in DSS-treated mice compared to mice that were not challenged with DSS. Although the authors did not profile microglia in these studies, these findings are consistent with the microglia hypothesis of Parkinson’s disease progression. However, it is unknown precisely how peripheral immune responses reprogram microglia. Illuminating the nature of this neuromodulation will be vital to develop strategies to protect the central nervous system from the pathological impacts of peripheral immune activation by the microbiome.

### Adaptive immunity: T lymphocytes in neuro-immune-microbiota communication

Immune cells in the meningeal compartments are essential for maintaining neurological homeostasis, including regulating behavior and resolution of neuroinflammation after injury [61]. T lymphocytes are critical coordinators and effectors of both mucosal and systemic immunity. Intestinal dendritic cells constantly sample the luminal contents of the gut, resulting in persistent priming of T cells by gut bacteria that primarily results in regulatory T cell (Treg) expansion [62, 63]. Thus, commensal microbes profoundly impact intestinal T cell activation and differentiation. Microbial metabolites, such as SCFAs, maintain immune homeostasis by inducing Treg differentiation [64, 65] (Fig. 2). Conversely, butyrate can activate antigen-specific CD8 + T cell populations, promoting anti-pathogen immunity in the gut [66]. Other metabolites that can alter T lymphocyte functionality include ascorbate (induction of T cell apoptosis) [67], mevalonate and dimethylglycine (inhibit the development of IFNγ + CD8 T cells) [68], and poly-γ-glutamic acid (induction of regulatory T cells), to name just a few [69]. Specific gut bacteria can also induce defined T cell subsets, such as Th17 induction by segmented filamentous bacteria (SFB) and Treg induction by *Bacteroides fragilis* [70–73].

Microbiota-induced peripheral T cell dysregulation can potentially lead to alterations in T cell populations in the CNS. For example, microbiota-induced increases in Th17 differentiation contribute to maternal immune activation–induced behavioral abnormalities [74] and exacerbate the mouse model of multiple sclerosis experimental autoimmune encephalomyelitis (EAE) [75] [76]. By combining single-cell RNA-sequencing and repertoire sequencing of Th17 T cells across peripheral and central tissues, a recent study solidified the connections between peripheral and CNS T cells in EAE. This study identified two subsets of Th17 cells in the CNS: a homeostatic SLAMF6 + population and a pathogenic CXCR6 + population that migrates to the central nervous system in EAE [77] (Fig. 2). Homeostatic SLAMF6 + Th17 cell populations in intestinal tissues gave rise to pathogenic CXCR6 + Th17 cells, which were significantly reduced in mice treated with antibiotics. These data suggest that microbial composition and density play a critical role in maintaining SLAMF6 + Th17 cells [77]. The conversion of SLAMF6 + to CXCR6 + Th17 cells was also reduced in antibiotic-treated mice [77], providing an attractive mechanistic explanation for the resistance of germ-free mice to EAE.

While SFB-induced Th17 cells can promote EAE, *B. fragilis*–induced Tregs can ameliorate EAE [78] (Fig. 2). The *B. fragilis* zwitterionic capsular polysaccharide A protects against severe inflammation in EAE by inducing the conversion of naive T cells to IL-10 + FoxP3+ regulatory T cells [79]. Although the exact mechanism of protection is unclear, it is postulated that these Tregs directly combat IL-17a induction. Recent studies describe a new role for the gut microbiota in the cross talk between peripheral and CNS immunity. Using paired single-cell and TCR repertoire sequencing, Papparlardo et al. found that the majority of T cells in human cerebrospinal fluid exhibit features characteristic of peripheral T cells, suggesting that peripheral T cell populations play a significant role in CNS immunity [80]. Furthermore, Benakis et al. found that bacterial dysbiosis suppressed effector T cell trafficking from the gut to the leptomeninges after acute ischemic brain injury [81]. Finally, the recent discovery that T cells inhabit the meninges both at homeostasis and during inflammation raises a myriad of new questions about the nature of this surveillance and its implication for central nervous system health and disease [82].

In addition to classical T cells, innate-like lymphocytes may also influence neuronal functions. For example, the release of IL-17a by γδ T cells in the meninges can directly activate cortical glutaminergic neurons and induce anxiety-like behaviors [61]. The gut microbiome also has a profound impact on the selection and function of invariant natural killer T (iNKT) cells [84]; thus, NKT cells may also form an additional link between the gut and the brain [85].

### Adaptive immunity: B lymphocytes in neuro-immune-microbiota communication

Accumulating data indicate that B cells also play a critical role in regulating CNS immunity. As with T lymphocytes, microbial metabolites can shape B lymphocyte differentiation and function (Fig. 2). *Lactobacillus*-derived 3-idoleacetic acid and lipopolysaccharide (LPS) enhance the production of IL-35 by Bregs [86]. SCFAs influence plasma cell differentiation and antibody production [87, 88], where acetate induces IgA production and butyrate suppresses IgA production [89, 90]. In the intestine, IgA+ plasma cells critically regulate microbiota composition and barrier function [91], and IgA production by intestinal plasma cells is rapidly induced following microbial colonization [92]. IgA levels in the meninges also depend on microbial colonization status. Like T cells, gut-derived plasma cells can also traffic to the brain [93]. Although IgA+ plasma cells are abundant in the dural venous sinuses of conventional mice, only very low levels of IgA+ plasma cells are found in the dural sinuses of GF mice. Colonization of ex-GF mice with human fecal microbes restored IgA+ plasma cell populations in the dural venous sinuses; notably, dural B cell repertoires overlapped with intestinal plasma cells, suggesting that dural venous sinus plasma cells originated in the gut [93]. IgA-producing plasma cells in the dural venous sinuses provide critical protection against infection as depletion of meningeal IgA+ plasma cells resulted in infiltration of fungi to the brain after intravascular injection [93].

Finally, several recent studies have highlighted immunoregulatory roles for gut-derived IgA+ plasma cells in the central nervous system. Early evidence of immune suppression by B cells emerged in 2010 when Lloyd Casper’s group found that antibiotic treatment of EAE mice induced expansion of CD5+ B cell populations in lymphoid organs and adoptive transfer of these CD5+ B cells conferred protection against EAE pathology [94]. More recently, microbiota-specific IgA+ plasma cells were shown to protect against EAE by trafficking to the central nervous system [95], presumably as part of a homeostatic response to reduce inflammation. This protection is mediated by gut-derived IgA+ plasma cells, which can traffic to the central nervous system and reduce disease severity in an IL-10-dependent manner [96]. Together, these studies show that the impacts of microbiota-mediated education of the immune system extend beyond the intestine to the CNS in both health and disease.

### The potential role of vagal and central neurons in neuro-immune-microbiota connections

The vagus nerve can serve as a physical conduit that directly relays signals from the gut microbiota to the central nervous system (Fig. 2). The vagus nerve comprises 80% afferent fibers and 20% efferent fibers [97], and vagal nerve endings in the gastrointestinal tract can sense luminal inputs [98, 99]. Numerous microbial-derived metabolites have been shown to impact vagal activation. The microbiota-derived SCFA oleate activates the vagus nerve via the CCK-A receptor [100], and butyrate can directly activate vagal afferent terminals in the gut [100]. Furthermore, vagal fibers express pattern recognition receptors such as Toll-like receptor 4, enabling direct detection of and activation by bacterial products [101]. Interestingly, targeted vagal stimulation can suppress LPS-induced pro-inflammatory cytokine production by microglia [102, 103]. The vagus nerve’s role in the gut-brain axis is commonly studied using vagotomized humans and mice. For example, vagotomized humans had a significantly lower risk of developing Parkinson’s disease [104, 105]. In mice, *Lactobacillus rhamnosus*–induced amelioration of anxiety- and depression-related behaviors was diminished after vagotomy [106, 107].

The vagus nerve is also thought to serve as a physical transporter of protein aggregates in Alzheimer’s and Parkinson’s disease via a mechanism analogous to prion disease. The accumulation of α-synuclein aggregates in gut vagal endings often precedes CNS symptoms in progressive Parkinson’s disease, and recent studies have shown that α-synuclein aggregates injected into the gut can transit to the brain via the vagus nerve [108] (Fig. 2). Gut bacteria can also produce amyloid proteins, such as curli or CsgA, which is important for biofilm formation and epithelial adhesion [109, 110]. Colonization with *E. coli*–producing curli exacerbated motor deficit in a mouse model of Parkinson’s disease [111], suggesting that microbial influences on α-synuclein aggregation in the gut can seed or enhance disease progression via the trafficking of peripheral protein aggregates to the central nervous system.

Recent evidence also suggests that microbial metabolites can directly influence the CNS (Fig. 2). For example, the circulation of microbial metabolites to the brain can affect thalamic axonogenesis in early life [112]. Also, specific microbial-derived metabolites associated with neuropsychiatric disorders, such as 3-(3-hydroxyphenyl)-3-hydroxypropanoic acid (HPHPA), are more abundant in cerebrospinal fluid from conventional mice as compared to GF mice [113]. Finally, recent pioneering studies underscore the bidirectional nature of the gut-brain axis (Fig. 2). Koren et al. demonstrated that a subset of neurons in the insular cortex were activated by DSS-induced intestinal inflammation [114]. Remarkably, reactivation of these neurons after removal of DSS led to the re-induction of intestinal inflammation, suggesting that these neurons can store and retrieve past immunological activation. Prior observations of psychosomatic immune responses, including conditional allergic responses, indicate the potential generalizability of CNS-mediated recall of past immune responses. Future exploration of the memory and recall capacity of the brain in neuro-immune-microbiota cross talk promises to further solidify this fascinating concept.

## Conclusion

Scientific and clinical endeavors to understand the gut-brain axis over the past three centuries have evolved in fits and starts toward the modern holistic approach that underlies the explosion of new interest in this area. The emergence of neuroimmunology as a core discipline that links the study of the nervous and immune systems has critically enabled our current understanding of the gut-brain connection. Furthermore, the technology-fueled explosion in microbiome research over the past two decades has ushered in a new era of exploration of the microbiota-gut-brain axis. Going forward, holistic approaches and close collaborations between normally disparate disciplines will be critical to expose the core mechanistic principles that underlie the complex and multi-dimensional interactions between these systems.
